# The Beginning of Meiosis in Mammalian Female Germ Cells: A Never-Ending Story of Intrinsic and Extrinsic Factors

**DOI:** 10.3390/ijms232012571

**Published:** 2022-10-20

**Authors:** Donatella Farini, Massimo De Felici

**Affiliations:** Department of Biomedicine and Prevention, University of Rome Tor Vergata, 00133 Rome, Italy

**Keywords:** meiosis, PGC reprogramming, retinoic acid, DAZL, MVH, meiotic cell cycle, STRA8

## Abstract

Meiosis is the unique division of germ cells resulting in the recombination of the maternal and paternal genomes and the production of haploid gametes. In mammals, it begins during the fetal life in females and during puberty in males. In both cases, entering meiosis requires a timely switch from the mitotic to the meiotic cell cycle and the transition from a potential pluripotent status to meiotic differentiation. Revealing the molecular mechanisms underlying these interrelated processes represents the essence in understanding the beginning of meiosis. Meiosis facilitates diversity across individuals and acts as a fundamental driver of evolution. Major differences between sexes and among species complicate the understanding of how meiosis begins. Basic meiotic research is further hindered by a current lack of meiotic cell lines. This has been recently partly overcome with the use of primordial-germ-cell-like cells (PGCLCs) generated from pluripotent stem cells. Much of what we know about this process depends on data from model organisms, namely, the mouse; in mice, the process, however, appears to differ in many aspects from that in humans. Identifying the mechanisms and molecules controlling germ cells to enter meiosis has represented and still represents a major challenge for reproductive medicine. In fact, the proper execution of meiosis is essential for fertility, for maintaining the integrity of the genome, and for ensuring the normal development of the offspring. The main clinical consequences of meiotic defects are infertility and, probably, increased susceptibility to some types of germ-cell tumors. In the present work, we report and discuss data mainly concerning the beginning of meiosis in mammalian female germ cells, referring to such process in males only when pertinent. After a brief account of this process in mice and humans and an historical chronicle of the major hypotheses and progress in this topic, the most recent results are reviewed and discussed.

## 1. Introduction

Meiosis is a special type of cell division unique of the germ cells of sexually reproducing species implying paternal and maternal genomic recombination and resulting in the production of haploid gametes that at fertilization fuse to reestablish normal diploidy in the zygote. It involves one round of DNA replication followed by two rounds of cell divisions, resulting in the mammalian male in four haploid germ cells, termed spermatids, and in the female in one mature oocyte and three small cells, termed polar bodies. Meiosis comprises two stages, based on the two rounds of divisions: meiosis I and meiosis II. Each stage can be subdivided into prophase, metaphase, anaphase, and telophase. Prophase I comprises events exclusive for meiosis and can be subdivided into four stages: leptotene (chromosome condensation), zygotene (the pairing of homologous chromosomes through the synaptonemal complex), pachytene (the initiation of the recombination between chromosome pairs and the formation of chiasmata between homologues at the crossing-over sites), and diplotene (chromosomes begin to separate, but remain attached via chiasmata). 

### 1.1. The Beginning of Meiosis in Mouse and Human Ovaries

In the mouse embryo, from around 10.5 days post coitum (dpc), the precursors of germ cells, termed primordial germ cells (PGCs), coming from the yolk sac enter into the developing gonadal ridges (GRs) and are soon subject to sex-specific signaling processes. At this stage, PGCs are sexually bipotential and can commit to either the spermatogenic or the oogenic pathway. In the developing ovaries, between 12.5 and 16.5 dpc, germ cells, still termed PGCs or oogonia, downregulate the expression of master pluripotency genes such as *Pou5f1* (also known as *Oct4*), *Sox2*, and *Nanog*, and upregulate or begin to express in an anterior-to-posterior wave throughout the ovary germ-cell-specific and meiotic genes such as *Dazl*, *Stra8*, *Mvh* (*Vasa or* *Ddx4*), *Sycp3*, and *Dmc1* [1,2].

The completion of genome-wide reprogramming and the expression of RNA-binding protein (RBP) DAZL sign germ cells’ competence to enter meiosis [3,4,5]. This relies on multiple signals, comprising retinoic acid (RA), the active metabolite of Vitamin A, diffusing from the ovary–mesonephros on germ cells and activating in the latter a broad gene expression program primarily involving *Stra8*, a crucial meiotic gatekeeper [6,7,8,9,10]. The first morphological evidence that female mouse PGCs enter meiosis is seen around 13.5 dpc, as they, now termed primary oocytes, begin to condense chromatin into chromosomes and assembly the synaptonemal complex. The loading of SYCP3, a synaptonemal complex protein, and REC8, a meiotic cohesin protein [11], onto the chromosomes marks the beginning of meiotic prophase I (MPI). The expression of genes such as *Spo11*, a topoisomerase that causes DNA double-strand breaks (DSBs), and *Dmc1*, involved in the formation of DSBs and homologous recombination, are associated to this event. Following the formation of DSBs, H2A histone family member X (γH2AX) is phosphorylated and recruited at the site of DNA breaks. In the developing testes, PGCs, also termed prespermatogonia or gonocytes, maintain the expression of pluripotency genes, continue to proliferate, and asynchronously enter G1/G0 mitotic arrest between 13.5 and 14.5 dpc.

In human fetal ovaries, early histological observations indicated that meiotic entry is initiated asynchronously from around 8 to 9 weeks post fertilization (GW = gestational weeks 10–11) and constitutes the transition from oogonia to oocytes [12]. This is confirmed by a marked increase in the expression of RNA-binding proteins DAZL and MVH (both at the transcript and protein levels) between weeks 7 and 12 [13,14] and the presence of γH2AX- and SYCP3-positive oogonia in week 8 and from weeks 10 to 12 [15], respectively. *STRA8* expression is detected and peaks around this developmental time; then, it remains detectable until weeks 16–18. The expression of *STRA8* is paralleled or followed shortly after by the upregulation of *SYCP3*, *SPO11*, and *DMC1*, which are indicators of meiotic progression [16]. In line with this notion, the expression of *SYCP3* in the ovaries of 8–10-week embryos and of *DMC1*, *SPO11*, and *SYCP1* from week 11 onwards [15,17] were reported. Because meiotic entry in humans is asynchronous, some oogonia initiate meiosis and differentiate into oocytes, while others continue to express pluripotency markers and remain proliferative until around weeks 17–18. This proliferating subpopulation of oogonia is mainly localized in the periphery of the ovaries, whereas the more differentiated oocytes are deeper within the medulla. After week 18, the majority of oogonia initiate meiosis, and oocytes arrest in the diplotene stage of the first meiotic division, where they are surrounded by pregranulosa cells forming single-layered primordial follicles [15,17].

### 1.2. Main Historical Steps in Understanding the Beginning of Meiosis in the Fetal Ovary

Histological studies describing meiotic oocytes in the fetal ovaries of several mammalian species, including humans, date from the first years of the 20th century until the 1960s and are referred to for the most part in the milestone paper by Baker [18]. Similar studies made clear that in testes of the same fetal age, germ cells do not enter meiosis but undergo several mitotic cycles before becoming quiescent within the forming seminiferous cords.

Following such morphological studies, it was proposed that germ cells of both sexes at the same time receive stimuli that induce the onset of meiosis [19] but that the somatic cells in the developing testis inhibit meiosis from proceeding [20,21]. 

According to this theory, Anne Grete Byskov interpreted the results of several in vitro cultures of mouse embryonic gonads carried out throughout the 1970s as a demonstration of the existence of a meiosis-inducing substance (MIS), likely produced by the adjacent mesonephros in both sexes, and of a meiosis-preventing substance (MPS) only in the fetal testes [22]. Many years later, naturally occurring sterols, termed meiosis-activating sterols (MASs), acting as MISs were identified [23]. In light of the recent findings concerning the crucial role of RA as a meiotic inducer (see next sections), the significance of MASs that share nuclear receptors related to those of RA, termed RORs, for the beginning of meiosis should be reinvestigated.

In the 1980s, Anne McLaren, while studying germ-cell sex differentiation in chimeric mouse fetal gonads with germ and somatic cells of opposite sexes or germ cells cultured outside the gonads in vitro, concluded that “*the decision to embark into oogenesis or spermatogenesis does not depend on their own chromosomes but only on their environment*” [24]. She supported the hypothesis that the fetal testis produces an inhibitor of meiosis but favored the possibility that the stage of fetal development at which meiosis begins in the germ-cell lineage is programmed. 

In this view, female germ cells enter meiosis in the fetal ovary and develop as oocytes following a default pathway, or if an MIS exists, it is present ubiquitously. Observations indicating that mouse female PGCs were inhibited to enter meiosis when aggregated with somatic cells from fetal testes in vitro [25] and that PGCs of both sexes began meiosis outside the gonads within the embryo [26] or outside the embryo in a simple cell culture system [27] corroborated these hypotheses. The following years were characterized by the search for extrinsic and cell-intrinsic drivers of meiotic initiation, a hunt that is still ongoing.

In the first decade of 2000, several studies reported that RA is an extrinsic factor exerting the MIS function in the mouse fetal ovary as well as in the testis. Conversely, its degrading enzyme, CYP26B1, acts as an MPS in the fetal testis [7,8,28,29,30,31]. From then, numerous studies confirmed the involvement of RA in the beginning of meiosis in both sexes in several other species, including humans [15,17]. At the same time or soon after, molecular studies and genetics models identified the *Stra8* gene as the principal RA target in meiosis-competent germ cells [2,6,7,8,9,10]. In 2011, Kumar et al. were the first to challenge the role of RA as an MIS, reporting *Stra8* expression in fetal ovaries from mice lacking RA synthesis (*Raldh*^-/-^) in mesonephros and gonads [32], thus proposing that RA is not the only inducer of *Stra8* expression and meiosis. 

Subsequently, numerous other papers aimed to identify molecular pathways, besides that driven by RA, involved in meiosis entry, followed and will be mentioned in the next sections. 

Currently, the advent of methods to generate in vitro PGCLCs from pluripotent stem cells [33,34,35] and single-cell RNA sequencing (scRNA-seq) analysis [36,37] promises the dissecting of the precise dynamics of gene expression during the beginning of meiosis both in model animals and in humans. 

The results obtained so far, some of the most significant reported in sections of the present review, represent a powerful way to confirm and extend previously acquired notions and uncover new players in this unique process.

## 2. Molecules and Conditions for Meiotic Beginning

### 2.1. Intrinsic Factors

**Figure 1:**
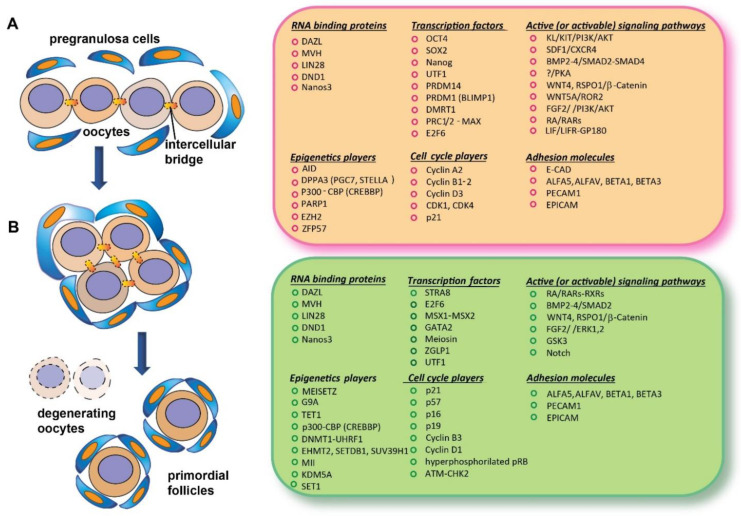
Schematic representation of an oogonium nest before entering meiosis (**A**) and of oocytes at the beginning of meiosis associated with nest breakdown and the formation of primordial follicles (**B**); on the right, the main classes of molecules identified in mouse oocytes during these developmental stages, with most of them being discussed in the present review.

The beginning of meiosis in germ cells requires the acquisition of a molecular status, critically including epigenomic and specific RNA-binding proteins, referable to what early researcher conceived as “intrinsic factors” (features not directly traceable to the action of specific external signaling) and a variety of local signals and conditions constituting the “extrinsic factors” (Figure 1).

#### 2.1.1. Genome-Wide Reprogramming in Premeiotic PGCs 

Early studies revealed that, in both sexes, the DNA of mouse PGCs settled in GRs was strikingly hypomethylated and that this epigenetic erasure involved imprinted genes [5,38]. Today, we know that this unique epigenetic status is part of a global genome-wide reprogramming proper of the preceding germline and probably necessary for the beginning of meiosis.

The most accredited aims of this reprogramming are apparently contradictory. It forms the basis to generate extremely specialized female and male germ cells but, at the same time, to set up in them a genomic status sufficiently plastic to rapidly regain, at fertilization, totipotency in the zygote and blastomeres. To learn more about this crucial process, readers can refer to [39].

We briefly discuss below the reprogramming in PGCs mainly as a function for the acquisition of the capacity to undertake meiosis in female germ cells.

PGC genome reprogramming relies on epigenetic modification, including global and locus-specific DNA demethylation and histone replacement, and post-translation modifications.

To these epigenetic processes, the recruitment of inhibitory and activatory complexes on specific gene loci are superimposed. The biological outcomes of these processes are multiple. They prevent the inheritance of acquired epigenetic information across generations and erase parental imprints so that they can be re-imposed in a sex-dependent manner. In addition, they reactivate loci on the inactive X in female germ cells and probably establish histone marks, making it possible to rapidly regain totipotency in the zygote and blastomeres.

In mouse PGCs, global and locus-specific DNA demethylation occurs in two subsequent phases [40]. The first stage happens in specified PGCs during their proliferation and migration towards GRs (7.5–10.5 dpc) [41,42].

Active and passive mechanisms of DNA demethylation work in concert. The first is likely mediated by Activation-induced cytidine deaminase (AID) and perhaps by Poly-(ADP-ribose) polymerase 1 (PARP1) [43,44,45]. The second one is related to DNA replication occurring during PGC proliferation in the absence of key components of the methylation machinery [41,46]. In this phase, transcription factors such PRDM1 (or BLIMP1) and PRDM14 (SOX17 in humans) [47] repress somatic transcriptional programs, while genes encoding core pluripotency transcription factors such as OCT4, SOX2, and Nanog, and germ-cell-specific genes (i.e., *Nanos3*) are activated [48].

Notably, during this phase, methyltransferase DNMT1 preserves DNA methylation in the differentially methylated regions (DMRs) of some imprinted genes, cytosine guanine island (CGI) promoters on the inactive female X chromosome, and of germ-cell-specific and meiotic genes [49]. Repressive histone marks H3K9me2, H3K27me3, and H2A/H4R3me2s, together with H2A.Z, and active histone marks H3K9ac, H3K4me2, and H3K4me3 contribute to refining the chromatin status of pre-gonadal PGCs [5,50,51,52]. In addition, catalytically active Polycomb repressive complex ½ (PRC1/2) was identified in 12.5 dpc PGCs. These chromatin remodelers are required to repress meiotic genes, *Stra8* included, from depositing repressive histone marks on their promoters [53].

The second reprogramming occurs after PGCs enter the GRs (11.5–13.5 dpc). The loss of repressive histone marks likely favors an open chromatin status [5]. As noted, among the repressive histones, the decrease in H3K27me3 is followed by an increase from 12.5 to 16.5 dpc concomitant with the beginning of meiosis in female germ cells [54], suggesting that a high level of this histone may be necessary for meiotic entry. Notably, histone modifications seem to be, at least in part, sex specific [55]. Interestingly, mouse PGCs obtained from 11.5–13.5 dpc specimens of both sexes revealed H3K4me3/H3K27me3 bivalent domains to be highly enriched at the developmental regulatory genes in a manner remarkably similar to that of embryonic stem cells (ESCs) [56]. Bivalent modification can contribute to the repression of somatic cell lineage genes and/or ready these genes for future activation.

Notably, locus-specific DNA demethylations, including germ-cell-specific and meiotic genes, occur in PGCs of both sexes so that most of the sequences previously maintained methylated by DNMT1 are demethylated [5,41,57]. Critically, this induces the expression of *Dazl* and others germ-cell-specific genes, such as *Mvh* and *Sycp3* [57,58].

TET1/2, the enzyme capable of converting 5-methylcytosine (5mC) into 5-hydroxy-methylcytosine (5hmC) [59], plays a critical role in these second demethylation round [57,60,61], perhaps with the participation of PARP1 [44]. TET1 seems, however, mostly devoted to the removal of aberrant residual and/or de novo DNA methylation and to the activation of germ-cell-specific and meiotic genes via a DNA demethylation-independent mechanism [61,62]. Interestingly, DAZL, which is essential for licensing gametogenesis (see below), associates with the mRNA of *Tet1* in mouse ESCs supporting its translation [63].

Collectively, these data suggest that one purpose of the epigenetic reprogramming in PGCs, besides those reported at the beginning of this section, is to ensure the timely and efficient activation of genes enabling progression towards gametogenesis and meiosis.

In such a view, epigenetic modifications might represent, at least in part, the hypothesized intrinsic factors governing the beginning of meiosis in female germ cells (see above). In supporting this notion, the ATP-dependent SWI/SNF remodeling chromatin complex counteracting PRC-mediated repression is required for the transition from sexually undifferentiated mouse PGCs to female or male germ cells [64]. In addition, Hill et al. identified a subset of genes activated during PGC epigenetic reprogramming, which are referred to as the Germline Reprogramming Responsive (GRR) genes that are activated after the depletion of 5mC and PRC1 in both male and female PGCs at E13.5 [62].

In the mouse embryo, following initial inductive triggering by growth factors such BMPs and WNT3a needed for germline specification inside epiblast cells [65,66,67,68,69], epigenetic reprogramming proceeds in the PGC precursors and subsequently in PGCs, provided that growth factors/cytokines sustain their proliferation and survival during migration and the colonization of GRs [70,71]. Understanding if and how signaling from the above pathways and environmental cues modulate the chromatin-modifying enzymes represents a challenge for future research.

Interestingly, recent studies demonstrated that metabolic changes also contribute to PGC reprogramming, since many enzymes that play important roles in epigenetic gene regulation utilize substrates generated via cellular metabolism [72,73]. Clearly, PGC reprograming is also associated with proliferation, and its completion implies a certain number of mitotic cycles. As reported above, at the end of these cycles (5–6 in the mouse, [74]), the demethylation-dependent expression of some genes, such as *Dazl* and some meiotic genes, specific histone marks in the regulatory regions of genes, and chromatin-remodeling proteins constitute the basis to enter meiosis in both females and males. In the fetal testis, however, early meiotic genes are rapidly silenced, whereas in the developing ovaries, their expression is amplified and followed by other middle and late meiotic genes under the induction of multiple signals from the somatic environment; this is discussed in the next sections.

A progressive downregulation of the expression of pluripotency genes such as *Oct4*, *Sox2*, *Nanog*, and *Lin28* is also critically required for the beginning of meiosis in female PGCs/oogonia [75].

Promoter deacetylation probably favors the downregulation of some of these master pluripotency genes, such as *Oct4* and *Sox2* [76], while the repression of the translation of their mRNAs is one function of DAZL and probably other factors [77] (see below). At the same time, active histone mark H3K4me3 decreases in the promoters of pluripotency genes and increases in those of meiotic genes [78]. DPY30 and ASH2L, components of a SET1-Like multiprotein histone methyltransferase complex, favored by RA (see Section 2.2.2, Retinoic Acid), are likely to catalyze H3K4me3 at the promoters of meiotic genes such as *Stra8*, *Rec8*, and *Sycp3* [75]. Moreover, KDM5a, a member of the histone demethylase family that can specifically remove dimethyl and trimethyl groups (H3K4me2/3) from the fourth lysine of histone H3, may be involved in the erasure of H3K4me3 at the promoters of pluripotent and cell-cycle genes [75]. All these epigenetic factors may play important roles in switching off pluripotency genes and switching on meiotic genes. In some of the latter, for example, *Stra8*, promoter acetylation also contributes to switching on expression [79].

#### 2.1.2. DAZL and MVH

Substantial evidence supports the notion that in mice and probably in all mammalian species, RBP DAZL is critical for rendering female PGCs/oogonia competent to enter meiosis within the fetal ovary and for sex-specific differentiation events of oogenesis and spermatogenesis [80,81,82]. Despite this, the gametogenic functions of DAZL have not yet been fully characterized. DAZL was localized in both the nucleus and cytoplasm of fetal germ cells [83]. As reported in the previous section, its expression is epigenetically regulated by the DNA methylation of CpG islands present in its promoter region. Moreover, the fact that the first exon and intron of the gene are highly enriched in the active histone H3K4me3 mark and lack repressive H3K27me3 [84] suggests that promoter demethylation can rapidly lead to gene activation. The expression of the *Dazl* gene, however, might also depend on signals from the gonadal ridges, since in the absence of these, PGCs of both sexes were shown not to undergo gametogenesis and to retain their pre-gonadal characteristics [58]. *Dazl* and *Mvh* (see below) are not likely direct targets of RA, one of the major extrinsic factors triggering meiosis in mammalian germ cells, since they were not reported to possess Retinoic Acid Response Elements (RAREs) in mammals. Intriguingly, however, a functional Retinoic Acid Receptor beta (RARβ)-binding RARE sequence (see Section 2.2.2, Retinoic Acid) was found in the chicken *Dazl* gene core promoter region [85].

Some findings suggested that RNA-binding protein NANOS3, known to be required for the maintenance of mouse PGCs during migration via the suppression of apoptosis [86], either directly or indirectly positively regulates the expression of the DAZL gene in human PGCs [87].

Deleted in azoospermia, the DAZ family consists of three members, DAZL, DAZ, and BOULE, which are exclusively expressed in pluripotent stem cells and germ cells [88]. As RBPs, these factors elicit their roles through modulating the translation and stabilization of specific mRNAs [89]. For example, DAZL, either directly, through 3′-UTR, or indirectly, through polyA-binding protein PABP, binds the mRNAs of germ-cell-specific genes, such as *Mvh*, *Sycp1*, *Sycp3*, *Tex11*, and *Tex14*, and facilitates the translation of their coded proteins [89,90]. Interestingly, as indicated above, *Tet1* was proved to be dependent on DAZL for its translation in cultured mouse ESCs [63].

Conversely, DAZL may exert a repressive role in mRNA translation. In fact, in PGCLCs, it targets several pluripotency genes (including *Sox2* and *Sall4*), represses their expression at the post-transcriptional level, and associates with the mRNAs of key caspases, inhibiting their translation and favoring germ-cell survival [77].

Notably, in mouse embryos, depending on the genetic background, the ablation of *Dazl* causes a severe reduction in germ-cell numbers and the aberrant expression of markers of pluripotency in post-migratory PGCs. Moreover, these cells show the inability to differentiate along the oogenesis or spermatogenesis pathway and in female germ cells to enter or progress through meiosis [4,80,81].

By profiling gene expression in mouse fetal ovary mutants, Soh et al. recently reported that DAZL is required for the induction of nearly all 104 genes that they identified to be specifically expressed during meiotic prophase [91], but the way in which it controls such transcriptional program remains to be clarified. A study documented that GASZ, a protein with four ankyrin repeats encoded by an evolutionarily conserved gene expressed exclusively in germ cells, interacts with DAZL and synergistically stimulates PGCLCs to form mouse ESCs [92].

Evidence exists indicating that DAZL also plays a crucial role in human oogenesis [93]. In the human embryo, the percentage of germ cells in the fetal ovary highly expressing DAZL was found to be increased markedly from 28 to 48% from weeks 10 to 18 of development, while that of OCT4-positive cells was found to be decreased significantly. Notably, cells expressing a high level of DAZL almost always lacked OCT4 expression, indicating a mutually exclusive expression pattern of the pluripotency marker and DAZL [94].

Moreover, a combination of DAZL and BOULE could be utilized to induce human ESCs to exit the pluripotent state and enter meiosis in vitro [94]. The identification of RNA codifying genes involved in chromosome cohesion and DNA recombination as targets of human DAZL (*SYCP3* and *TEX19*, for example) highlights the importance of this RBP also in early meiosis [95].

Similar to *Dazl*, *Mvh* (also known as *Vasa* or *Ddx4*) is expressed in germ cells around the time of PGC arrival at the gonadal ridges and requires the gonad environment for expression [96].

Although *Mvh* expression is independent of DAZL [58], the latter is able to bind *Mvh* RNA and modulate its translation [90]. Moreover, as reported above, *Mvh* expression is partly dependent on demethylation [42].

MVH is an ATP-dependent RNA helicase that often changes the secondary structures of RNA during processes such as alternative splicing and protein translation initiation [97]. MVH interacts with ribonucleic acids through its conserved DEAD (Asp-Glu-Ala-Asp)-RNA-binding motif and exerts multiple roles in the germline of various species [98]. For example, it appears to be able to regulate the proliferation and pluripotency of PGCs and meiosis in male germ cells; however, no specific functions of the protein in mammal oogenesis were found. Indeed, the loss of *Mvh* in the mouse affects the number and differentiation of male germ cells but apparently not oogenesis [99].

### 2.2. Extrinsic Factors

**Figure 2:**
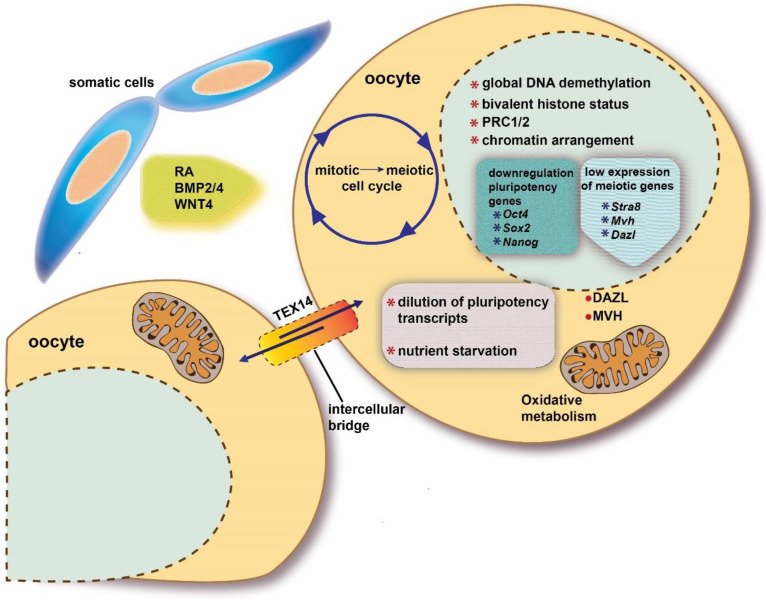
Schematic representation of intrinsic and extrinsic factors discussed in the present review involved in the beginning of meiosis in a fetal oocyte.

Once a specific epigenetic status and the expression of DAZL RBP are established, PGCs become responsive to the action of local ovary/mesonephros factors and conditions that though various intracellular molecular cascades, promote or inhibit in the developing ovaries and testes, respectively, their entering into meiotic prophase I. Such extrinsic factors cause a mitotic G0 block in male PGCs/prospermatogonia, and a switch from the mitotic to meiotic cycle and the full activation of meiotic genes in female PGCs/oogonia (Figure 2).

#### 2.2.1. The Switch from Mitotic to Meiotic Cell Cycle

The in vitro transdifferentiation of isolated premeiotic PGCs into pluripotent stem cells, termed EG cells, by specific cocktails of growth factors (KL, FGF2, LIF) or GSK3 and MAPK inhibitors (2i) [100] and the formation of Embryonal Carcinoma (EC) cells in vivo from undifferentiated PGCs [101] confirm the essential influence of signals from the fetal gonad environment on directing the correct germ-cell differentiation and meiosis. Moreover, these occurrences offer some clues on the processes that critically control the timely entering of PGCs into meiosis in fetal ovaries and into mitotic arrest in fetal testes. Among these there are the already mentioned epigenetic genome status and the downregulation of pluripotency genes associated with the activity of intracellular pathways depending on external signaling demonstrated to drive, at least in vitro, PGC proliferation/survival. These include KL/KIT-dependent PI3K/AKT pathways, PKA and ERK1/2 pathways, and PKCε, a novel DAG-dependent PKC. The manipulation of PGC epigenetics (i.e., the inhibition of deacetylation) or of signal transduction pathway components (i.e., the constitutive expression of AKT and the addition to the culture medium of FGF2, FRSK, PKCε inhibitor, or 2i) prevents the beginning of meiosis and makes both female and male PGCs prone to transdifferentiate in proliferating EG cells [100]. Interestingly, most of these molecular pathways seem to be present and active in human PGCs during the transition to premeiotic oogonia [102].

In the next sections, we discuss how some signals identified in the last years in the fetal ovary can inhibit or redirect these pathways in the context of the entering into meiosis.

Scarce information is available on cell-cycle molecules involved in the switch from mitosis to meiosis. We know that mouse PGCs take the decision to enter meiosis rather than continue mitosis in G1 before pre-meiotic DNA synthesis [6]; however, proteins critical for the beginning meiosis must be present and active in G2 preleptotene.

Regarding the G1 phase, early studies by Western et al., 2008, showed that in female mouse premeiotic PGCs, the critical regulator of mitotic G1–S transition, pRB, was present but became hyperphosphorylated and inactive, while another member of the RB family, *Rbl1*, was downregulated [103]. More recent studies in mouse and human germ cells revealed that the transcripts of other players in the mitotic G1–S transition, such as *Ccnd1*, were low in mitotic germ cells and sharply increased in early meiotic cells, whereas *Ccna1* was exclusively present in meiotic germ cells [104]. The expression of *Ccna2* and *Ccnd3*, both also exerting a role in the G2–M transition, was restricted to mitotic germ cells and downregulated in meiotic prophase. *Cdk1* and *Cdk4* transcripts appeared to be restricted to mitosis, while *Cdkn2a* (p16) and *Cdkn2d* (p19), inhibitors of cyclin D/Cdk4/Cdk6 complexes, increased consistently with *Ccnd1* in early meiotic germ cells.

Conversely, *Cdkn1a* (p21) and *Cdkn1c* (p57), inhibitors of most cyclin/Cdk complexes, decreased during the transition from mitosis to meiosis. Whether some of these changes contribute to meiosis entry or are a consequence of the change in the cell division process remain to be established.

In the G1 phase of the cell cycle, many DNA replication regulatory processes begin. In the case of meiosis, DNA replication during the S phase produces pairs of sister chromatids, held together by cohesin complexes. Much of our understanding of this pre-meiotic DNA replication comes from studies using yeasts, which reported several differences between mitotic DNA replication and pre-meiotic DNA [105,106,107]. The pre-meiotic S phase is also of longer duration than the mitotic S phase in mammals (from two to three times) [108,109,110]. This substantial extension of the pre-meiotic S-phase is still largely unexplained, although the synthesis of proteins such as STRA8, DMC1, SYCP3, Hormad1, and REC8, needed for setting up the inter-homologous relationships important for the recombination and correct segregation of homologous chromosomes, is a possible reason. Recently, STRA8, as well as its germ-cell-specific interactor, Meiosin, emerged as the protein required for the proper expression of many meiotic cell-cycle genes [111,112]. We discuss the involvement of STRA8 and Meiosin in the mitosis/meiotic switching operating in this phase in another section below.

The analysis of key G2/M cell-cycle proteins revealed that entry into MPI involves the repression of G2/M-promoting *Cyclin B1/B2*, coincidently with the upregulation of repressing *Cyclin B3* and the robust establishment of the key checkpoint ATM/CHK2 and ATR/CHK1 pathways [113]. In this phase, RNA-binding protein MEIOC, upregulated at the onset of meiosis in both female and male mouse germ cells, was proposed to maintain an extended meiotic prophase stabilizing meiotic RNAs, such as that of *Ccna2*, through its interaction with YTHDC2 [114]. Germ cells lacking *Meioc* initiate meiosis; they undergo pre-meiotic DNA replication and express proteins involved in synapsis and recombination, and a subset of cells progress as far as the zygotene stage of MPI. However, cells in early meiotic prophase proceed to condense their chromosomes and assemble a spindle, as if having progressed to metaphase [115]. Thus, MEIOC, by preventing the premature exit from MPI, would allow the dynamic chromosomal program to be completed.

#### 2.2.2. Retinoic Acid

As anticipated in the previous section, multiple signals from the somatic cells of the gonad–mesonephros region are necessary for correctly promoting meiosis. Despite some criticisms (see above and [32,116,117,118]), several in vivo and in vitro studies indicated that RA is involved in triggering or permitting meiosis in a paracrine manner in mammal germ cells of both sexes [119].

In the signaling cells, the conversion of retinol to RA requires two sequential oxidative steps, catalyzed by retinol or alcohol dehydrogenases (RDHs or ADHs) and by retinaldehyde dehydrogenases (RALDHs), respectively. Cytochrome P450 enzymes CYP26A1, CYP26B1, and CYP26C1 finely control the level of RA present in each tissue, balancing its synthesis and degradation [120].

In the target cells, RA serves as a ligand for two families of nuclear receptors, the RA receptors (RARs) and the retinoid X receptors (RXRs). The RA/RAR/RXR complex binds to RAREs in the target genes, recruiting corepressors or coactivators and consequently producing transcriptional changes. In addition, in certain cell types, RA was reported to activate the PI3K and ERK1/2 MAPK signaling pathways through a rapid RAR-dependent non-genomic mechanism that does not require new gene transcription or newly synthesized proteins [121]. Moreover, retinol was found to affect cell fate through cell membrane receptor STRA6, which does not function merely as a retinoid transporter but is also able to affect cell fate through other pathways, such as those involving p53, JAK/STAT, Wnt/β-Catenin, and calcium [122]. Finally, RA regulates the expression of many miRNAs [123].

As far as we know, however, no information is available about non-genomic RA action, STRA6 receptor presence, and miRNAs regulated by RA in germ cells.

All the components of the classical RA system are consistently present in the right place and at the right time in the ovary–mesonephros of embryos of various mammalian species, including humans, and when the system is perturbed, meiosis is usually impaired. Moreover, Vitamin A deficiency in vivo was reported to prevent meiosis initiation in the fetal rat ovary [124], while the addition of exogenous RA was shown to accelerate the initiation of meiosis in organ cultures of rodent and human ovaries [8,15,17]. Interestingly, oogonia present in human embryonic ovaries within 5 weeks from fertilization did not initiate meiosis in response to RA treatment but rather increased their proliferative activity [125], thus indicating that the lack of expression of a meiotic facilitator renders oogonia unresponsive to RA or that a currently unidentified meiotic inhibitor may exist in such early embryonic ovaries. A similar stimulation of proliferation by RA on mouse pre-gonadal and early gonadal PGCs was reported [126,127]. Clearly, PGCs/oogonia acquire the competence to interpret RA as a MIS following entry in the GRs. The complete molecular basis of such “license” is not clearly defined, but as reported above, the epigenetic status of chromatin and DAZL functions is crucially involved.

In mice, a flux of RA responsible for the beginning of meiosis in the fetal ovaries is believed to come mainly from the mesonephros between 11.5 and 14.5 dpc [7]. A local ovary contribution to RA production is also possible [128,129]. A rostro-caudal wave of expression of genes such as *Dazl*, *Stra8*, *Sycp3*, and *Rec8* is typically described in mouse female germ cells between 13.5 and 15.5 dpc, whereas that of pluripotency markers is quenched in the same direction [1,2]. In fact, the expression of a large number of genes is modulated in the transit of PGCs to primary oocytes in the mouse ovary between 12.5 and 15.5 dpc. As reported above, this includes pluripotency and meiotic prophase genes, and RA likely plays a crucial role in modulating both classes of genes.

The finding that RA plays a role in the repression of the PGC program, which involves a network of TFs for pluripotency [130], is consistent with a well-known function of RA in stem-cell differentiation [131]. As for meiotic genes, Soh et al. [91] identified 100 out 104 meiotic prophase genes upregulated by RA in DAZL-expressing female germ cells. Besides *Stra8*, which we discuss below and in the next section, these include *Rec8* and *Smc1b*, required for chromosome cohesion; synaptonemal complex assembly genes such as *Sycp1*, *Sycp2*, and *Sycp3*; genes for chromosome synapsis and the formation of DSBs, such as *Me1*, *Msh5*, *Dmc1*, and *Hormad1*; but also 50 so far uncharacterized proteins.

Of note, this transcriptional program characterizing MPI beginning in the ovary is very similar to that engaged by preleptotene spermatocytes [111,112], and it was highlighted that the expression of more than 50% of them is STRA8 dependent [42,91,112].

In the human ovary, meiosis initiation probably requires intrinsic RA synthesis, which is ensured by ALDH1 expression in somatic cells closely surrounding oogonia [15,17]. As in the mouse, the expression of a large number of genes is regulated in the transit of oogonia to primary oocytes between 11 and 19 weeks post fertilization [132].

In both mice and humans, *Stra8* is considered a major RA target in germ cells entering meiosis. How RA induces *Stra8* expression is, however, still the object of debate. As reported above, in the classical model of RA-dependent gene activation, unliganded RAR–RXR heterodimers bind to RARE sequences and repress the transcription of their associated genes, unless activated by RA binding. Additional co-regulators and epigenetic changes critically contribute to transcriptional regulation mediated by RA. All such components are probably involved in the RA-dependent stimulation of *Stra8* expression. Studies in mice indicated that *Stra8* possesses classical RARE sequences, but its involvement in *Stra8* activation by RA is not completely clear [133,134].

Numerous results suggested that the regulation of *Stra8* relies, at least in part, on acetylation and methylation changes at the level of its promoter. For example, in F9 teratocarcinoma cells, histone deacetylases (HDACs) repressed its transcription, whereas HDAC inhibitor TSA maximized it [135]. Furthermore, in ESC cells, the most prominent binding sites for CTCFL (BORIS), a key coordinator of the three-dimensional chromatin structure, localize to the first intronic region of *Stra8*, and when CTCFL is overexpressed, *Stra8* is induced. Of note, in the adult mouse testis, CTCFL distribution in germ cells overlaps completely with that of STRA8, suggesting that it serves to induce or maintain its expression [136].

In line with these observations, data obtained from mouse ESCs demonstrated that RA was able to regulate *Stra8* expression through CBP (or CREB1 binding protein), which possess intrinsic histone acetyltransferase (HAT) activity. RA signaling recruits CBP to the RARE sequences present in the *Stra8* promoter, which in turn enhances the level of AcH3 and AcH4 and the recruitment of RNA polymerase II to activate *Stra8* expression [79].

In the mouse, the germ-cell-specific deletion of DNA methylase enzyme DNMT1 revealed that the *Stra8* promoter is normally methylated and repressed for several days after PGCs colonize the fetal gonad and that in the absence of the enzyme, germ cells undergo precocious meiotic entering [49]. Feng et al. recently reported that in mouse female PGCs, Notch signaling is probably necessary for the acquisition of the demethylated status of the *Stra8* promoter necessary for its activation by RA [137]. A chromatin-based gene regulation of the *Stra8* promoter is also suggested by the observations that in mouse female germ cells, *Stra8* resides in a bivalent chromatin region and is a direct target of RNF2, a component of the PRC1 complex that suppresses *Stra8* transcription. RA was shown to remove the repression imposed to the PRC1/2 gene shortly before meiosis began in the fetal ovaries [53], ensuring the correct timing of *Stra8* elevation [53]. Noticeably, in mouse ESCs, MAX, a protein component of atypical PRC complex PRC1.6, was reported to be responsible for the repression of *Stra8* and other meiotic genes as well as meiotic onset. Moreover, the expression level of MAX in female PGCs entering meiosis was extremely low and was much higher in male PGCs, which do not begin meiosis but undergo mitotic arrest [138].

Finally, in mouse ESCs, RA was reported to be able to activate germ-cell and meiotic genes, including *Stra8*, in an indirect way by relying on the BMP-SMAD1/5 pathways [139]. In line with this, RA and BMP2/4 were shown to increase *Stra8* expression in the mouse fetal ovary through transcription factor MSX1, which in F9 cells, was able to bind to *Stra8* regulatory sequences [140].

Considering such results, but also those casting doubts about the fact that RA is crucial for the beginning of meiosis [116,117,118,141,142], it is plausible instead is that RA is not the only factor controlling *Stra8* activation and, almost certainly, not the only extrinsic factor involved in regulating the beginning of meiosis in the fetal ovaries. In supporting this last notion, the ablation of CYP26A1, a very potent RA-degrading enzyme, did not impair the formation of STRA8-positive cells but decreased *Stra8* transcription [7], thus indicating that CYP26B1 has other activities apart from metabolizing RA in fetal gonads and suggesting a role of endogenous RA in amplifying *Stra8* expression, rather than being the Initial inducer of the gene.

Other extrinsic signals and molecular pathways appear to collaborate with RA to regulate *Stra8* expression and meiosis entering, as studies aimed to elucidate the mechanisms of how STRA8 drives meiotic initiation, discussed in the next section, revealed.

#### 2.2.3. STRA8

*Stra8* was first identified in P19 teratocarcinoma cells as a RA-inducible gene and erroneously described as being restricted to male germ cells in fetal testes [143].

Gene knockout studies in mice clearly demonstrated that *Stra8* is required for meiotic initiation and the meiotic progression of germ cells and that the ablation of the gene results in infertility in both sexes [6,7,8,9,10]. In mouse female embryos, *Stra8*-deficient PGCs do not initiate meiotic chromosome condensation, cohesion, and synapsis, or DNA double-strand breaks and recombination [6].

**Figure 3:**
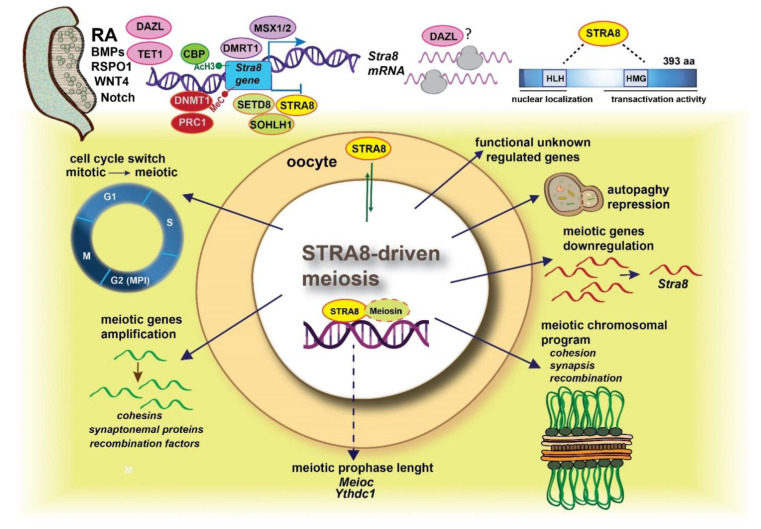
Schematic representation of the main factors so far detected that control the expression of the *Stra8* gene and of the functions attributed to the STRA8 protein as transcription factors. For details, see text.

As reported in the section above, *Stra8* expression is crucially dependent on the promoter epigenetic status, and RA stimulates *Stra8* expression though multiple mechanisms involving both RAREs and epigenetics. However, over the years, other factors involved in the activation or repression of the gene were identified. Among the first, there are DMRT1 and MSX1/2, and among the second, SOHLH1, SETD8, and STRA8 itself (Figure 3).

Krentz et al. [144] reported that in the mouse fetal ovary, the most part of the germ cells lacking transcription factor *Dmrt1* showed a greatly reduced expression of *Stra8* and during MPI fail to properly localize synaptonemal proteins SYCP3 and γH2AX involved in DNA repair. Experiments of chromatin immunoprecipitation combined with mRNA expression profiling suggested that the transcriptional activation of *Stra8* is the main function of DMRT1 in mouse female germ cells and that this regulation is direct via binding to sequences proximal to RARE motifs. Since *Dmrt1* expression in mouse and human fetal ovaries showed a similar transient expression pattern, peaking at meiotic entry [145], it seems plausible that DMRT1 is also required for the initiation of meiosis in human female germ cells.

Intriguingly, in mouse male adult germ cells, DMRT1 exerts an opposite role, since it inhibits meiosis entry by blocking DNA-dependent *Stra8* transcription [146].

Le Bouffant et al. [140] reported that mouse fetal ovaries from double *Msx1*^-/-^ and *Msx2*^-/-^ mice showed significantly decreased *Stra8* expression at 14.5 dpc but not at 13.5 dpc, thus suggesting that MSX proteins are not required for the initial induction of *Stra8* expression but rather for maintaining or increasing its expression [140]. In human fetal ovaries, MSX transcription factors are expressed around the period of the beginning of meiosis, suggesting their involvement in the first stage of this process also in our species [147].

WNT4 and RSPO1, other growth factors similar to BMPs of the TGFβ family, might also take part in *Stra8* activation through unknown pathways. In fact, the expression of *Stra8* resulted to be downregulated in the partially masculinized *Wnt4*-deficient mouse ovary [148], and in the absence of *Rspo1*, a proportion of PGCs neither expressed *Stra8* not entered meiosis [149].

Desimio et al. [150] found that SOHLH1 and SOHLH2, two germ-cell-specific basic helix-loop-helix (bHLH) transcription factors, were able to directly bind to the *Stra8* promoter through two canonical E-Box motives and cooperatively to repress its expression in vitro. Considering the expression timing of these transcription factors and *Stra8* in mouse fetal ovaries, the authors suggested that they cooperate with other transcription factors to ensure *Stra8* downregulation in the mid–end stages of MPI rather than to play a role in the beginning of meiosis.

In addition, SETD8, the only known monomethyl transferase of histone 4 at lysine 20 (H4K20me1), and an important regulator of the G1–S transition, binds directly to the proximal promoter of *Stra8* and inhibits its expression [151]. In spermatogenesis, this action, associated to the repression of *Setd8* expression mediated by STRA8 itself and a parallel formation of SETD8-STRA8 complexes [152], seems to be involved in the complicated regulation of spermatogonium proliferation/differentiation and entering meiosis [151], but whether these interaction occur in the ovary is not known.

Since it could be important that STRA8 is expressed transiently at the beginning of meiosis, this regulation is also probably ensured by a negative feedback operated in oogonia by STRA8 itself [91], which is able to bind its promoter [91,111,112].

Given for a fact the crucial role of STRA8 in the beginning of meiosis, results from recent papers began to shed light on how STRA8 drives meiotic initiation (Figure 3). The first study attempting to characterize the molecular function of STRA8 reported that the protein shuttles between nucleus and cytoplasm but that it was mostly nuclear in freshly isolated germ cells [153]. In addition, protein–DNA cross-link studies showed that STRA8 is able to bind DNA and displays transcriptional activity when fused to a GAL4-DNA-binding domain [153,154]. Furthermore, STRA8 possesses putative bHLH and HMG box domains, implying its role as a DNA-binding protein [6,112,153] Effectively, STRA8 is involved in the transcriptional gene program that characterizes the meiotic entry both in female and male gonads, as evidenced by the profiling of transcriptome in fetal *Stra8*^-/-^ ovaries [91], in preleptotene spermatocytes [112], and *Stra8*^-/-^ testes [111]. This program includes both downregulated and upregulated genes, and surprisingly, most of them are expressed in early preleptotene spermatocytes that express low *Stra8* levels and have not engaged MPI yet [112]. It can be concluded that STRA8 amplifies a transcriptional program rather than inducing it. STRA8-dependent genes include those involved in meiotic chromosome dynamics, such as cohesins (e.g., *Stag3*, *Smc1b*, and *Rad21l)*, synaptonemal complex assembly (e.g., *Sycp2-Syce1-3*), or recombination (e.g., *Dmc1*, *Msh5*, *Hormad1*, etc.). Of particular relevance is the observation that genes involved in the cell cycle are regulated by STRA8, including the transcriptional regulators of the G1/S transition, such as *E2f1* and the *cyclins/Cdk* complex.

Additionally, different transcriptional (e.g., *Pparg)* and post-transcriptional regulators (e.g., *Meioc* and *Ythdc2*) involved in the control of meiotic prophase length (see above) are STRA8-regulated [91,111,112], thus indicating that other factors could participate in the STRA8 regulation of the meiotic program. In fact, how STRA8 exercises its transcriptional control is not completely clear. It is able to directly bind the genomic regulatory region close to the transcriptional start site (TSS) of the regulated genes [111,112] at a consensus motif (the CNCCTCAG sequence) that does not correspond to a E-Box sequence recognized by bHLH transcription regulators [155].

The same consensus region in the most meiotic-regulated genes is shared by Meiosin, a recently discovered STRA8 interactor in prospermatocytes [111]. This protein is also expressed in the mouse fetal ovary at the same time as STRA8, and ovarian germ cells fail to start meiosis when *Meiosin* is ablated as in *Meiosin*^-/-^ spermatocytes. In addition, Meiosin, which possesses bHLH and HMG DNA-binding domains, similar to STRA8, controls a large transcriptional program, including meiotic and non-meiotic genes, most of which are also STRA8-regulated, thus indicating that Meiosin and STRA8 act in concert to amplify the network, ensuring the exact gene level indispensable for the beginning of meiosis at the right time. Do they conduct this by binding to open chromatin and recruiting RNA polymerase II, releasing it, or via other mechanisms? This is still an open question.

By comparing the expression of the 104 genes that characterize ovary MPI in *Kit^W^*-, *Stra8-*, and *Dazl*-deficient mice, Soh et al. [91] considered the expression of about half of these genes (including *Dmc1*, *Hormad1*, *Me1*, *Msh5*, *Rad21l*, and *Me1ap*) to be fully dependent on STRA8, while that of the remaining was considered to be partially dependent (i.e., *Sycp1*, *Sycp2*, *Sycp3*, *Spo11*, *Stage3*, and *Smc1b*) or, in a few cases, largely independent (i.e., *Rec8*). RA regulates both STRA8-dependent and -independent pathways. The other classes of meiotic genes partially dependent or completely independent from STRA8 would also or only require for expression RA/RAR/RXR complexes, respectively.

Despite such a central role of *Stra8* in the initiation of meiosis, early meiotic genes, as mentioned above, are expressed in germ cells of both sexes some time before *Stra8* when they are still in mitosis [156,157]. Thus, this suggests that STRA8 likely binds open chromatin regions and functions to amplify transcriptional levels rather than inducing the transcription of unexpressed genes. Fully or partially STRA8-independent genes are probably induced earlier to prepare cells for the meiotic chromosomal events later triggered by the transcription factor. According to Soh et al., [91], these genes include cohesins and synaptonemal complex proteins. Early induction probably also involves genes encoding proteins controlling the mitotic-to-meiotic switch, since in female embryos lacking *Stra8* gene function, PGCs fail to undergo premeiotic DNA replication [6]. Moreover, STRA8 directly activates *E2f1*, which drives the G1–S cell-cycle transition [158], and as reported above, in male germ cells, STRA8 directly upregulates the genes encoding post-transcriptional regulators MEIOC and YTHCD2, which are critical for the unique meiotic cell-cycle status. Finally, STRA8 regulates the expression of *Setd8*, interacts with its encoded protein (an important regulator of G1-S transition), and regulates *Ccne2* and *Cdk2* genes, considered “general” cell-cycle genes.

Although STRA8 seems to exert a transcription factor role by mainly upregulating meiotic gene expression, repressive functions on non-meiotic genes are also evident [111,112]. In a recent study, STRA8 was shown to interact with itself and with other bHLH transcription factors through its HLH domain, and it was noted that this domain was important for its ability to negatively interfere in vitro with the E-Box-mediated transcriptional activity of bHLH transcription factors, among which are SOHLH1 and SOHLH2 [150].

### 2.3. Other Factors

A number of factors, some known to play crucial role in the beginning of meiosis in other species, but little considered till now, are emerging as novel players in this process. Some of these factors exert a negative action that must be removed, while others corroborate the inductive actions of RA discussed above to assure an efficient and correct beginning and progression of MPI.

#### 2.3.1. Inhibitory and Cooperative Factors

The downregulation of the FGF9 and NODAL growth factors is considered as a prerequisite to make meiosis possible in the mouse embryonic ovary. FGF9, expressed in the somatic cells of both male and female undifferentiated GRs, remains highly expressed only in fetal testes, specifically in pre-Sertoli cells, mainly exerting a paracrine action on germ cells [29,159]. Conversely, NODAL, a member of the TGFβ superfamily, appears to be mainly expressed in PGCs and might act in an autocrine manner [137,160].

Intriguingly, in the human embryo, NODAL was found to be expressed in both female and male mitotic germ cells, whereas its receptor, ACVR1C, and target gene *Pitx2* were specifically expressed in female meiotic and male mitotic-arrest germ cells [161]. Nonetheless, at least in the mouse, the absence of either FGF9 or NODAL can be considered permissive for meiosis onset rather than inductive.

β-Catenin was also recently proposed to exert a negative action on the beginning of meiosis. Indeed, Le Rolle et al. [162] reported that β-Catenin was present in the nucleus of proliferating mouse PGCs physically associated with OCT4 and regulated chromatin accessibility to promoters and intergenic regions of several genes. The ablation of *β-Catenin* caused the exit from the mitotic cycle and the precocious expression of *Dazl* and meiotic genes including *Stra8*. In light of these and other results, the authors proposed that the activation of GSK3 and the upregulation of ZNRF3, an E3 ubiquitin-protein ligase that acts as a negative regulator of the WNT signaling pathway, in PGCs promote the inactivation of β-Catenin and contribute to the switch from mitosis to meiosis.

Conversely, Activin A might cooperate with RA to promote meiosis. When Activin A was added to cultured 12.5 dpc mouse ovaries or delivered to 10.5 dpc litters via intraperitoneal injection, the expression of *Stra8* and of meiotic genes in germ cells was increased. The activation mechanism was suggested to be indirect via the activation of SMAD3 in pre-granulosa cells and the downregulation of CYP26B1 [163].

As mentioned below, BMPs emerged as crucial partners of RA in the beginning and progression of MPI. Early studies in our laboratory showed that the addition of BMP4 to the culture medium of isolated mouse PGCs increased their proliferation rate and favored their entering into meiosis in the presence of RA [164,165]. BMP acts through ZGLP1, encoding a meiosis-specific transcriptional regulator with GATA-like zinc fingers that is able to switch on repressed bivalent genes, contributing to determine the oogenic fate and entering into meiosis [130,142], whereas RA signaling contributes to the repression of the PGC program and the overall maturation of the oogenic program [130]. Interestingly, mouse PGCLCs did not display the capacity for oogenesis when RA alone was added to the culture medium, while they did when BPM2 alone was added [142]. In line with this notion, quite surprisingly, some *Stra8*- and *Meiosin*-deficient mouse PGCs could grow and differentiate into oocyte-like cells that could be fertilized and produce a two-cell embryo unable of further development [111,166], while *Zglp1*^-/-^ female germ cells were unable to form oocytes and degenerate [112]. Interestingly, BMP signaling was gradually activated in human and monkey fetal germ cells entering meiosis, suggesting that it probably plays a role during the oogonium–oocyte transition in such species [161]. Importantly, BMP signaling was also proved to be crucial for the regulation of *Zglp1.*

Finally, Notch signaling was also proposed to play a role in meiotic onset. Indeed, it was shown that inhibitors of Notch signaling, or the siRNA-mediated gene knockdown of the protein, diminished the expression of *Stra8* and other meiosis-related genes in mouse fetal gonad cultures. This signaling, however, is more likely to promote the progression of oocytes through prophase I rather than the beginning of meiosis [137]. Interestingly, in human embryos, Notch signaling pathways seem to be activated in gonadal somatic cells by ligands expressed by neighboring germ cells and other adjacent somatic cells [161].

#### 2.3.2. Nutrient and Metabolic Factors

Nutrient restrictions and changes in metabolisms were recently proposed to act in concert with RA stimulation to activate the meiotic program [167]. This is reminiscent of meiosis initiation in yeast, which mainly depends on nutrients and metabolism.

The expression of transcription factor IME1 upon nutrient starvation in yeast triggers the activation of meiotic genes such as *SPO11*, *REC8*, *HOP1* (*Hormad1* in human), and *DMC1* [168]. In this species, autophagy is likely to be an important factor of meiotic initiation, as it is known to be activated upon nutrient deprivation [169].

Autophagy is crucial for reserving energy in response to cellular stress conditions such as nutrient and oxygen starvation. In both fission and budding yeast, meiotic entry fails if autophagy is deficient [170,171]. Autophagy might contribute to degrade major meiotic entry inhibitors, thus allowing the cell to enter meiosis [172]. However, in mammals, STRA8 represses autophagy by binding the promoter of NR1D1, which in turn leads to repressing its downstream target ULK1, an autophagy initiator, highlighting the requirement for the suppression of autophagy during meiosis initiation [173].

This means that although the role of nutrient deprivation in meiosis is potentially conserved, it is yet to be fully understood in mammals. It can be hypothesized that the suppression of autophagy via STRA8 might be important in maintaining meiotic DSBs during prophase I, as autophagy plays a role in DNA damage repair. The master regulator of autophagy, mTORC1, was found to be crucial during meiotic onset. In fact, in both yeast and female Drosophila, the reduction in TORC1 expression in response to nutrient starvation was needed for the mitotic–meiotic switch [174,175]. In mammals, the suppression of mTORC1 activators is required for male mitotic arrest in PGCs, which could prime male germ cells for meiotic entry [176]. Sahin et al. [177] demonstrated that mTOR is a target of RA signaling required for STRA8 expression during male meiotic onset. Together, these observations suggest that in mammals, meiotic initiation requires mTORC1 expression and thus the suppression of autophagy, as these two factors are mutually exclusive. Consequently, metabolic stresses that act on non-autophagic pre-meiotic cells, in parallel with RA stimulation, may be prerequisite for meiotic entry.

Based on scRNA-seq data, Zhang et al. reported that nutrient starvation is likely to be the metabolic stress inducing the switch from glycolysis to mitochondrial oxidative phosphorylation, as recently reported [167]. In the female ovary, this stress might be produced by germ-cell nests restricting nutrient access. Nest formation is a process where cysts form through rounds of mitosis of the oogonia without cytokinesis. Each nest is surrounded by several pregranulosa cells and contains several oogonia connected by intercellular bridges [178]. In *Drosophila* ovaries, these structures allow the transfer of oocyte-specific proteins, mRNAs, and organelles from supporting “nurse” cells to the dominant oocyte to be performed. In mammals, intercellular bridges play similar functions [179], and WNT4 seems to be involved in maintaining germ-cell cysts by providing a female pattern of E-Cadherin and β-Catenin expression within the germ cells [179]. Oogonia might cease cycling and synchronously enter meiosis within a cyst following the broad action of STRA8. The mechanisms that drive the breakdown of cysts and lead to the formation of primordial follicles are not known in detail. However, it is known that TEX14 is required for the formation, maintenance, and/or stability of the intercellular bridge both in male and female germ cells [180]. Recent results suggested a model alternative to the metabolic stress by which germ-cell nests could regulate the timing of meiotic entry across the ovary. This model states that the coordination of the mitotic–meiotic transition within a cyst depends on the stabilization of intercellular bridges mediated by TEX14. The authors observed that in the absence of *Tex14*, the levels of transcripts of pluripotency genes in some oogonia decreased more rapidly and meiosis began prematurely [181].

They proposed that cytoplasmic sharing may synchronize the pluripotent state of germline cysts by increasing the cytoplasmic volume, as meiotic transcripts must be expressed by a quorum to initiate meiosis. Asymmetries in gene expression arising among the cells within a nest would be diluted and equalized across intercellular bridges. In the absence of dilution, the levels of transcripts of pluripotency genes decrease more rapidly in some oogonia, which express meiotic transcripts and enter meiosis individually.

## 3. Conclusions

In the present review, we report old and novel results supporting a comprehensive model of the beginning of meiosis in the mammalian fetal ovary. According to this model, extrinsic factors such as RA and members of the TGFβ family, BMP2 in particular, secreted by cells of the ovary–mesonephros region send signals to the germ cells made responsive by intrinsic factors represented by a unique epigenetic status and the RNA-binding protein DAZL expression established in pre- and early gonadal PGCs/oogonia.

These factors first lead to the downregulation of pluripotency genes and the suboptimal activation of meiotic genes; then, with the essential contribution of RA- and BMP-dependent transcription factors STRA8 and ZGLP1, respectively, they lead to the amplification of a broad transcription program necessary to make possible/activate the meiotic cell cycle. The combined loss of DNA methylation and Polycomb complexes is required for germline-reprogramming responsive gene activation, with this epigenetically poised state further requiring TET1 and STRA8 to potentiate both full and efficient activation. STRA8, however, is able to amplify rather than induce the transcription program, including MPI genes, factors mediating the G1–S cell-cycle transition and DNA replication, and genes that promote the lengthy prophase unique to meiosis.

Probably, STRA8 is also able to repress pathways, such as those involved in autophagy, whose role in the beginning of meiosis awaits to be clarified, but that alone is not sufficient to drive a proper meiotic entry. Despite the progress in discovering new meiotic players, the entire molecular mechanism of meiotic initiation is still a partially solved question, as recent results about the crucial involvement of intercellular bridges, nutritional, and metabolic factors in the process demonstrated. In addition, the timing and regional differences in the beginning of meiosis in the fetal ovaries of experimental models and humans emphasize that several aspects of meiosis initiation in humans cannot be fully explained by current knowledge and that further studies are needed.

## Data Availability

Not applicable.
